# Quo vadis? *Philosophy, Ethics, and Humanities in Medicine *- preserving the humanistic character of medicine in a biotechnological future

**DOI:** 10.1186/1747-5341-4-12

**Published:** 2009-08-14

**Authors:** James Giordano

**Affiliations:** 1 Blackfriars Hall, University of Oxford, UK; 2 Center for Neurotechnology Studies, Potomac Institute for Policy Studies, Arlington, VA, USA

"*Philosophy is the sister of medicine*"

Tertullian

Over the past fifty years, the palette, depth and breadth of scientific discovery has advanced at amazing speed, and given the relevance of Moore's Law to applications of bio-computational capability, will likely continue, if not increase in pace. Yet, if we are to consider the information gained as more than merely knowledge for its own sake, then such knowledge must be regarded in terms of its potential uses and misuses in serving and affecting the human condition. I believe that this is especially true in medicine, what Edmund Pellegrino has called "...the most humanistic of the sciences, and most scientific of the humanities".

Toward such ends, the core domains (and tools) of philosophy - epistemology, anthropology, ethics, and to some extent metaphysics - play a formative and grounding role in analyzing, discussing and even determining the scope and tenor of medicine, as both a profession and practice. But perhaps, the task at hand is not to pose solutions, but rather to consider Bruno Latour's claim that science does not provide answers, but only raises additional questions, and in this way gain insight to what we know, how we know it, the ways in which such information and knowledge are used in applications of curing, healing, and caring (viz- medicine), and how skill, knowledge and art can -and perhaps should - be employed toward effecting the personal and public "good" that medicine professes to provide.

Realistically, we cannot extricate medicine from the social fabric in which it is enacted, nor is it logical to do so. Thus, it is important to acknowledge that our technological progress has fostered enhanced globalization, and in this way, medicine must engage an increasingly pluralized socio-cultural milieu, with diverse values, needs, and perspectives. This is the anthropological dimension of medicine, and in articulating practice - as best described by Alasdair MacIntyre as an exchange of good between agents in relationship - we must consider and appreciate the nature of such good, its meaning, and how these constructs affect the conduct of both clinical care and research.

Hence, medicine is intellectual, practical, therapeutic and moral, and therefore, any meaningful consideration of its practices must address the inherent moral questions, issues, and problems, and regard the numerous ethical systems and approaches that may viably provide the means toward potential resolutions. Obviously, any such discussion is varied and rich, reflecting the myriad viewpoints of those involved, and the diversity of factors that can, and often will affect the activities of medicine. Clarity of these viewpoints is vital, and so we must sharpen the lens through which we perceive and even predict medicine's role in a changing world culture. This lens consists of multiple facets, each affording a somewhat different light, and all being important to the overall spectrum of intention, causes and effects.

It is in this light that I view *Philosophy, Ethics and Humanities in Medicine*. A journal is both forum and nexus. In the former sense, it is a vehicle with which particular perspectives can be elucidated and shared. In the latter, it links these perspectives both to each other, and to others that lie beyond, as part of an iterative discourse. With this spirit, I invite authors from many disciplines to contribute to the lens afforded by *PEHM*, so as to bring incisiveness, parallax, and color to the views offered in these pages. Of course, the process of peer-review remains rigorous; in the spirit of collaboration such review is not exclusory, but rather assistive, such that I seek to enhance mentorship of those authors who wish to improve their work and sharpen its focus.

I seek to conjoin the Editors, each and all well-respected scholars from a variety of fields, to provide such guidance when and where needed and desired. As well, I call for a more pro-active participation of our team of Associate Editors to generate speculation, commentary and editorials about not simply those papers that appear in *PEHM*, but more broadly, so as to offer their erudition - and stimulate discussion - on current and provocative issues in the fields of medical philosophy, ethics and medical humanities, at-large. I urge our Associate Editors to develop thematic issues that bring together past and current papers focal to current and/or emerging topics that are of interest and value to contemporary medicine, and am equally interested in seeking creative input from our readers and authors.

As Editor-in-Chief, I accept a position established and defined by the work of my predecessors, Dan Stein and Michael Schwartz, who will remain with the journal as Founding Editors, Emeriti. Without doubt, I am stepping into big shoes, and with humility, look to stand upon their shoulders with a view toward the future. I view my role as mentor, guide, and sometimes commentator, enabling voices to be heard, lenses to be focused and weaving together ideas by cultivating contributors' scholarly work, reflections and insights. I also look to preserve and expand the journal's international reach. If medicine is to "go global", as called for by the World Health Organization and UNESCO (among others), then these viewpoints cannot be isolated, but must be shared in plurlalogue, that I hope will contribute to true dialectic.

Toward these ends, I strongly encourage authors working in non-developed, and developing countries to submit their writing - for your experiences, speculations, and orientations are important if medicine is to sustain its humanitarian value in this world of shifting populations, merging cultures, and polyglot ethics. Here, I believe, is where medicine and the humanities fuse, in a meaning best construed by the German word *Geisteswissenschaft *"the spirit of science as ways of knowing". To be sure, as we move forward it becomes evermore meaningful to preserve the humanities as the spirit of medicine, so as to enable the right use of scientific knowledge and biotechnological tools toward the fulfillment of good ends. For as William Osler presciently noted some hundred years ago, "...without the humanities, medicine loses a part of itself". I agree, and hope that *PEHM *will remain a strong antidote to such loss.

